# The NLRP3 and Pyrin Inflammasomes: Implications in the Pathophysiology of Autoinflammatory Diseases

**DOI:** 10.3389/fimmu.2017.00043

**Published:** 2017-01-27

**Authors:** Carlos de Torre-Minguela, Pablo Mesa del Castillo, Pablo Pelegrín

**Affiliations:** ^1^Unidad de Inflamación Molecular, Instituto Murciano de Investigación Biosanitaria-Virgen de la Arrixaca (IMIB-Arrixaca), CIBERehd, Hospital Clínico Universitario Virgen de la Arrixaca, Murcia, Spain; ^2^Unidad de Reumatología Pediátrica, Hospital Clínico Universitario Virgen de la Arrixaca, Murcia, Spain

**Keywords:** inflammation, NLRP3, pyrin, extracellular ATP, P2X7 receptor, cryopyrin-associated periodic syndrome, familial Mediterranean fever

## Abstract

Inflammasomes are multiprotein complexes that critically control different aspects of innate and adaptive immunity. Among them we could highlight the release of pro-inflammatory cytokines that induce and maintain the inflammatory response. Usually, inflammasomes result from oligomerization of a nucleotide-binding domain-like receptor (NLR) after sensing different pathogenic or endogenous sterile dangerous signals; however, other proteins such as absent in melanoma 2, retinoic acid-inducible gene I, or pyrin could also form inflammasome platforms. Inflammasome oligomerization leads to caspase-1 activation and the processing and release of the pro-inflammatory cytokines, such as interleukin (IL)-1β and IL-18. Mutations in different inflammasomes are causative for multiple periodic hereditary syndromes or autoinflammatory diseases, characterized by acute systemic inflammatory flares not associated with infections, tumors, or autoimmunity. This review focuses on germline mutations that have been described in cryopyrin-associated periodic syndrome (CAPS) for *NLRP3* or in familial Mediterranean fever (FMF) and pyrin-associated autoinflammation with neutrophilic dermatosis (PAAND) for *MEFV*. Besides the implication of inflammasomes in autoinflammatory syndromes, these molecular platforms are involved in the pathophysiology of different illnesses, including chronic inflammatory diseases, degenerative processes, fibrosis, or metabolic diseases. Therefore, drug development targeting inflammasome activation is a promising field in expansion.

## Danger Signals, Inflammasomes, and the Physiological Significance of the Inflammatory Response

Inflammation is the response of the innate immune system to a noxious stimulus, including infections or tissue damage (1, 2). Characterization of inflammasomes represents a considerable advance in the understanding of the inflammatory molecular events that occur in response to infections, and importantly, to tissue damage in the absence of pathogens. Furthermore, inflammasome activation has also been attributed to changes on physiological homeostatic parameters, such as changes in extracellular osmolarity (3, 4), and virtually, any perturbation in homeostasis could generate a local or systemic inflammatory response (1, 2). Tissue damage and alteration of the homeostatic parameters induce the release of danger signals from the cells that activate the inflammasome in innate immune cells (5). Danger signals are usually referred as danger or damage-associated molecular patterns (DAMPs). The dual use of the term “danger” or “damage” in the acronym DAMP denotes that danger signals are not only released after damaging conditions but also in response to dangerous situations, such as during cellular environment alterations. In homeostasis, cells in tissues are in a physiological “basal” state maintained by nutrients, oxygen, growth factors, and adherence to other cells and the extracellular matrix. Changes in environmental parameters (temperature, osmolarity, oxygen, or pH) induce a cellular stress response and the subsequent release of DAMPs. Stress is then recognized by tissue-resident macrophages, activating different signaling pathways, including inflammasomes, and inducing an inflammatory response aimed to restore tissue functionality during noxious conditions. This inflammatory response was termed para-inflammation by Medzhitov (1). Deregulation of para-inflammation is intimately related with immunity and involved in the pathogenesis of immune-mediated diseases, being the base for the chronic low-level inflammation associated, for example, to type 2 diabetes (6). If homeostasis imbalance continues or is complicated with infection, cells become necrotic inducing an acute inflammatory response that will damage the tissue (7).

Damage-associated molecular patterns are intracellular components released to the extracellular milieu in response to cell stress or necrosis that activates different inflammatory pathways, such as inflammasomes. Inflammasomes are multimeric complex of innate immune receptors, activating caspase-1 and proteolytic mechanisms involved in pro-inflammatory cytokines [interleukin (IL)-1β and IL-18] (8). During cell stress, plasma membrane becomes permeable to ions, such as K^+^, or to intracellular metabolites, such as the nucleotide adenosine triphosphate (ATP) or uric acid (1). One of the best characterized DAMP is ATP, since in physiological homeostatic conditions, ectonucleotidases maintain low extracellular ATP concentration, but during necrosis or inflammatory conditions, a high extracellular ATP concentration is reached, and the purinergic P2X7 receptor is activated in macrophages (9–12). P2X7 receptor is a potent activator of the inflammasome in macrophages and other innate immune cells (9). Leakage of cellular proteins with intracellular functions is another example of DAMPs; the release of these proteins usually follows secretory pathways independent of the endoplasmic reticulum (ER) and Golgi apparatus. Activation of caspase-1 by inflammasomes controls the release of these intracellular proteins by activating different unconventional release pathways, including a particular type of cell death called pyroptosis (1, 13, 14). Caspase-1 ultimately controls the release of inflammasome particles, a signal produced to amplify the release of DAMPs by activating caspase-1 in neighbor cells (11, 15). The high mobility group box 1 (HMGB1) nuclear protein is another example of DAMP released upon caspase-1 activation. HMGB1 presents histone-binding properties in the nucleus, and in the extracellular milieu, HMGB1 engages the advanced glycation end-product-specific receptor in conjunction with toll-like receptors (TLR) to induce an inflammatory response (16). In conclusion, innate immunity mechanisms converge in producing an inflammatory response as a consequence of infection, tissue damage, or loss of homeostasis.

## Inflammasome Sensor Proteins

**Figure 1:**
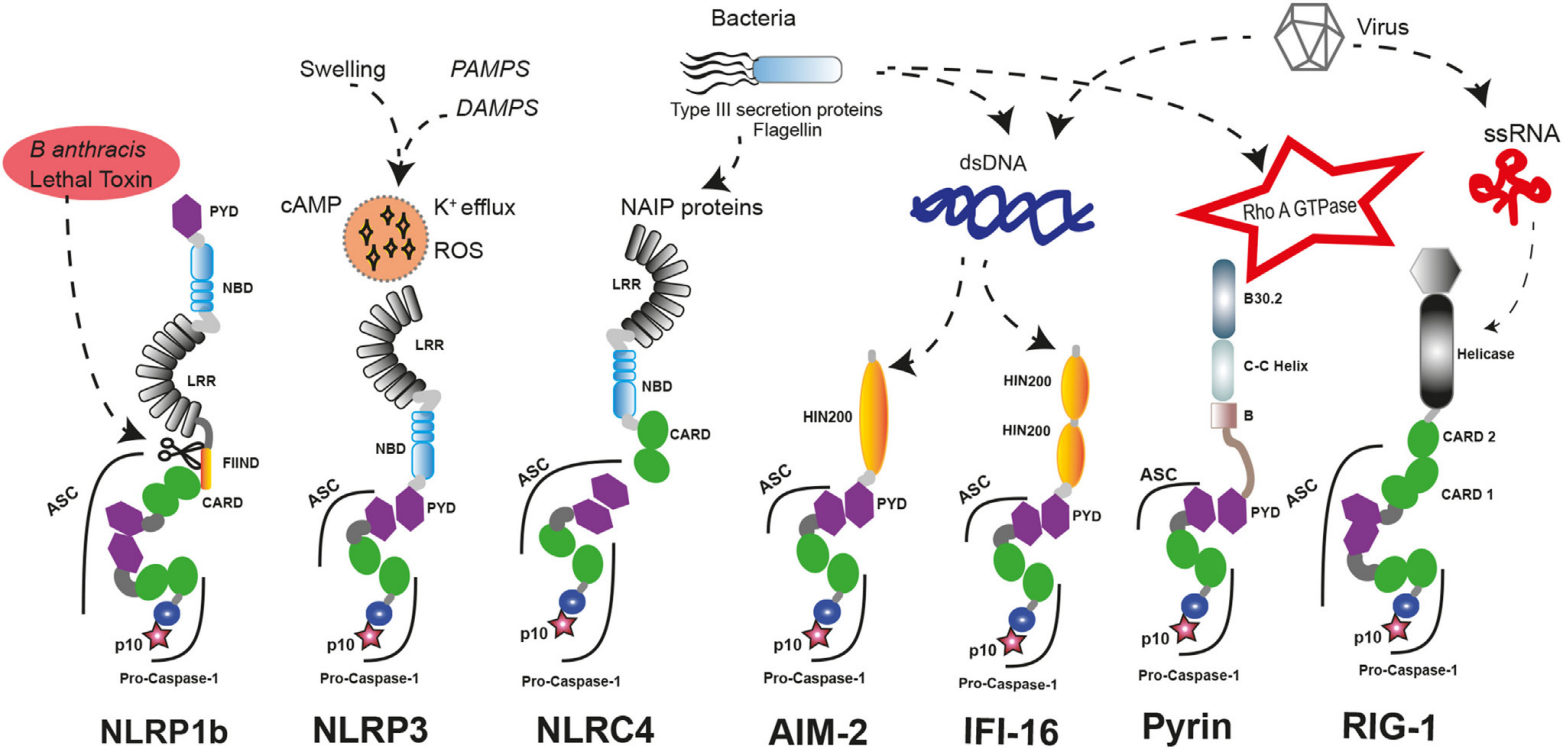
**Inflammasome sensors and activators**. A wide variety of pathogenic ligands and intracellular mediators are involved in inflammasome assembly. NLRP1b responds to proteolytic cleavage on their N-terminal induced by lethal toxin of *Bacillus anthracis*. NLRP3 is a general sensor of cellular damage that responds to intracellular harm induced by pathogenic or sterile insults. NLRC4 recognizes bacterial proteins *via* NLR family-apoptosis inhibitory proteins (NAIPs) and can assemble inflammasomes with or without recruiting ASC, similar to NLRP1b. Absent in melanoma 2 (AIM2) and interferon-inducible protein 16 (IFI-16) sense dsDNA through their HIN-200 domains; meanwhile, RIG-1 activates caspase-1 through an inflammasome assembly after it detects ssRNA. Pyrin inflammasome is induced by bacterial toxins that modify RhoA GTPase. DAMPs, danger-associated molecular patterns; PAMPs, pathogen-associated molecular patterns; ssRNA, single strand RNA, dsDNA, double strand DNA.

The nucleotide-binding domain-like receptor (NLR) family forms the main group of proteins considered as inflammasome sensors. These proteins contain a pyrin domain (PYD) or a caspase activation and recruitment domain (CARD). The presence of one of these domains in the sensor protein is required to assemble the inflammasome. Additionally, other proteins with some of these structural domains can also form functional inflammasomes, like absent in melanoma 2 (AIM2) protein, interferon-inducible protein 16 (IFI-16), retinoic acid-inducible gene I (RIG-I), and pyrin (17) (Figure 1).

There are different inflammasome sensors dedicated to recognize the presence of cytosolic nucleic acids. AIM2 presents an N-terminal PYD and a C-terminal hematopoietic interferon (IFN)-inducible nuclear protein with 200-amino acid repeat (HIN-200) domain. AIM2 is critical to respond against the infection of different pathogens by forming an inflammasome after recognition of double-stranded DNA (dsDNA) in the cytoplasm by the HIN-200 domain (18–20). Interestingly, other nucleic acid sensor protein called IFI-16 has two C-terminal HIN-200 domains and one N-terminal PYD. Upon detection of dsDNA, IFI-16 triggers the IFN response as a component of the signaling pathway (21) and can also induce the assembly of inflammasome with ulterior caspase-1 activation (22). RIG-I is also a sensor for viral RNA that contains two CARD domains and is able to assemble an inflammasome (23). However, it should be noted that additional studies are required to demonstrate that IFI-16 and RIG-I can form an inflammasome.

The structure of the sensor protein family NLR presents a central nucleotide-binding domain (NBD), and most of them have a C-terminal leucine-rich repeat (LRR) domain. The N-terminal protein domain is used to classify this group of proteins in NLRP if it contains a PYD domain or NLRC if it contains a CARD domain (24). Interestingly, the capacity for assembling inflammasome is a feature that has not been described for all members of the NLR family. These sensor proteins are also involved in other aspects of innate immune response by regulating diverse non-inflammasome pathways. Indeed, NLRP12 can play a role as a negative regulator of NF-κB signaling (25) or modulating IL-4 production in T cells (26), and NLRP6 is a negative regulator of mucosal immunity in the gut (27, 28).

The first sensor protein identified to form inflammasome was NLRP1 (29). Interestingly, human NLRP1 contains two additional protein domains compared to the canonical domains of the NLR family, such as a function-to-bind domain and a C-terminal CARD. These domains seem to play a critical role to assemble functional inflammasomes, as proteolytic cleavage of their N-terminal by pathogen components of *Bacillus anthracis* is required for their activation (30, 31). Furthermore, the presence of a CARD domain in the C-terminal allows the direct interaction and activation of caspase-1 without the presence of any other adaptor proteins like the apoptosis speck-like protein with a CARD domain (ASC), even though ASC incorporation to the platform enhances the processing of IL-1β (32), and in human THP-1 monocyte cell line, ASC is required for NLRP1 activation (33). In contrast, mouse NLRP1a could form an inflammasome independent of ASC (34).

A genetic study of families with vitiligo with or without other autoimmune diseases has revealed a link between these autoimmune disorders and the presence of polymorphisms in *NLRP1* gene (35). Recently, a novel gain-of-function mutation in *NLRP1* gene that predisposes to inflammasome activation has been associated with NLRP1-associated autoinflammation with arthritis and dyskeratosis autoinflammatory syndrome (36). This syndrome is characterized by diffuse skin dyskeratosis, autoinflammation, autoimmunity, arthritis, and elevated transitional B-cells (36) (Table 1). Furthermore, *NLRP1* mutations have been implicated in non-fever inflammasome-related disorders, in particular with two overlapping skin disorders: multiple self-healing palmoplantar carcinoma and familial keratosis lichenoides chronica, demonstrating that NLRP1 has an important role controlling skin inflammation (33).

**Table 1:** **Molecular and clinical features of autoinflammatory diseases associated with mutations in inflammasome sensor proteins**.

Disease	Disease symptoms	Clinical treatment	Inflammasome sensor	Gene	Mutations detected (references)	Mouse model (references)
CAPS	Systemic activation Urticarial rash CNS: deafness, cephalea, meningitis Musculoskeletal Amyloidosis	Anakinra^a^ Rilonacept^a^ Canakinumab^a^	NLRP3	*NLRP3*	(37–40)	(41–45)
FMF	Periodic fever Serositis/arthritis Myalgia Erysipeloid rash Amyloidosis	Colchicine^a^ Anakinra Canakinumab	Pyrin	*MEFV*	(39, 46–48)	(49, 50)
PAAND	Fever Neutrophilic dermatosis Myalgia/myositis	Anakinra^b^	Pyrin	*MEFV*	(51)	–
AIFEC	Early onset recurrent macrophage activation syndrome High levels interleukin (IL)-18	Dexamethasone^b^ Cyclosporine^b^ IL-18-binding protein^c^	NLRC4	*NLRC4*	(52–54)	(55)
CAPS-like syndrome	Cold triggered Arthralgia–myalgia Fever Urticarial rash	NSAIDS^b^ Anti-IL-1^b^	NLRP12	*NLRP12*	(56–58)	–
NAIAD	Recurrent fever Dyskeratosis Arthritis Metaphyseal abnormalities	Acitretin^b^ Anti-IL-1^b^	NLRP1	*NLRP1*	(36)	–
FKLC	Symmetric hyperkeratotic lichenoid papules	UVB phototherapy^b^	NLRP1	*NLRP1*	(33)	–
MSPC	Multiple recurrent keratoacanthoma Palmar-plantar-eye Risk of squamous cell carcinoma	Surgery^b^	NLRP1	*NLRP1*	(33)	–

*AIFEC, autoinflammation with infantile enterocolitis; CAPS, cryopyrin-associated periodic syndromes; FKLC, familial keratosis lichenoides chronica; FMF, familial Mediterranean fever; MSCP, multiple self-healing palmoplantar carcinoma; NAIAD, NLRP1-associated autoinflammation with arthritis and dyskeratosis; PAAND, pyrin-associated autoinflammation with neutrophilic dermatosis*.

*^a^Approved clinical treatment*.

*^b^Clinical treatment approach*.

*^c^Emergency compassionate-use Investigational New Drug authorization*.

The most prominent member of NLR family in the study of hereditary autoinflammatory syndromes is NLRP3. Indeed, gain-of-function mutations on *NLRP3* gene have been identified in patients with cryopyrin-associated periodic syndromes (CAPS, see below) (59, 60) (Table 1). NLRP3 contains the three canonical domains described in the NLRP family: PYD, NBD, and LRR, and it is able to assemble a functional inflammasome in response to a wide variety of triggers, suggesting that it could be a global sensor of cellular damage and different pathogens (5).

Besides NLRP3, formation of active inflammasomes triggered by a bacterial infection has only been described *in vitro* for other two members of NLRP family: NLRP7 (61) and NLRP12 (62). Interestingly, NLRP12 displays a sequence similar to NLRP3, and it is predominantly expressed in myeloid-monocytic cells (63). In some cases, genetic studies of symptomatic patients with CAPS-like syndrome without mutations in *NLRP3* revealed the presence of mutations in *NLRP12* gene (56, 57). *In vitro* study of these NLRP12 variants has shown an increase in the activity of caspase-1 and the secretion of IL-1β, suggesting the potential role of NLRP12 mutations in CAPS-like syndrome-associated inflammation (Table 1) (58, 64).

NLRC4 is another well-known member of the NLR family assembling functional inflammasomes in response to pathogens. NLRC4 is a component of a detection system for bacterial proteins such as flagellin and several components of the type III secretion system (65, 66). As a member of the NLRC subgroup, NLRC4 contains a C-terminal CARD besides of NBD and LRR domains, but unlike other NLR sensor proteins, NLRC4 requires of sensors co-receptors, termed NLR family-apoptosis inhibitory proteins (NAIPs), that recognize the pathogen proteins in the cytoplasm and oligomerize NLRC4 (67, 68). Similar to NLRP1, NLRC4 could interact directly with pro-caspase-1 through their CARD domain generating an inflammasome with a less efficient state of activation, and the association with the adaptor protein ASC is important to amplify the activation of caspase-1 (69). Gain-of-function mutations in *NLRC4* gene are associated with early onset autoinflammation with enterocolitis or recurrent macrophage activation syndrome depending on the mutation (Table 1) (52, 54). These patients are characterized by mutations in the NBD region of NLRC4 and benefits from recombinant human IL-18-binding protein therapy (53). The autoinflammatory-associated NLRC4 mutation H443P is able to constitutively activate caspase-8 and induce apoptosis *via* interaction with the component of the 26S proteasome Suppressor of Gal 1 and with ubiquitinated cellular proteins (70).

All inflammasome sensor proteins are activated in response to different pathogen and danger signals, suggesting that each activator triggers the formation of its own particular inflammasome complex. Interestingly, a recent work describes the recruitment of two sensor proteins (NLRC4 and NLRP3) to the same inflammasome complex as a result of the recognition of different danger signals from the same pathogenic infection (71).

Pyrin is another important inflammasome-forming protein (72). This protein contains an N-terminal PYD domain that is responsible for their interaction with ASC and later activation of caspase-1, a central coiled-coil domain and a C-terminal B30.2/SPRY domain that is not present in the mouse orthologous protein. The pyrin-inflammasome assembly could be triggered after sensing the activity of bacterial toxins from different species that covalently modify switch-I region of Rho family proteins (73). In addition, mutations in the gene that codify pyrin, *MEFV* gene, are found in symptomatic patients with hereditary autoinflammatory disorders (see below and Table 1) (46).

## Inflammasome Adaptor and Effector Protein Assembly

Inflammasome sensor proteins are involved in the recognition of particular danger stimulus and then initiate the assembly of inflammasome multimeric complex; in most inflammasomes, the interaction with an adaptor protein is required to enhance the activation of caspase-1. The protein ASC (also known as Pycard) is the ubiquitous adaptor for inflammasomes, and its interaction with the active inflammasome sensor protein induces a prion-like oligomerization process essential for the final structural conformation of the inflammasome. ASC is composed by two death-fold domains, a N-terminus PYD and a C-terminus CARD (74, 75). For those inflammasome sensor proteins associated with autoinflammatory disorders, i.e., NLRP3 or pyrin, their PYD domain is responsible for ASC recruitment *via* PYD–PYD homotypic interactions inducing the formation of filamentous structures that assemble into a large protein aggregate (76). Caspase-1 activation occurs within this aggregate, and interestingly, the same process of polymerization for ASC and pro-caspase-1 has been shown independent of the inflammasome sensor protein activated (77).

Recent works have provided additional information about the interactions between the components of the inflammasome, suggesting an initial self-nucleation of the sensor protein (NLRP3 or AIM2) promoting the assembly of helical ASC filaments *via* PYD homotypic interaction (78, 79). These ASC filaments, generated after multiple PYD interactions, expose CARD domains in the outer part of the filament and consolidate the inflammasome aggregation with an appropriated cross-linking between filaments *via* CARD–CARD interactions (80). The multiple oligomerization of pro-caspase-1 with the ASC filaments also occurs *via* CARD–CARD interactions and amplifies the danger signal started by the sensor protein (81).

## NLRP3 Inflammasome Activation Pathways

The activation of NLRP3 inflammasome appears in response to infection and is amplified by danger signals triggered during the infection, or by tissue injury or alterations in tissue homeostasis without infection. As it was described before, the majority of inflammasome sensor proteins are able to recognize different pathogen-associated molecules (bacterial proteins, toxins, and nucleic acids) and therefore activate inflammasome assembly in response to a microbial or viral infection. NLRP3 sensor is particularly able to oligomerize in response to a wide variety of stimuli that include pathogen molecules such as bacterial cell wall components or pore-forming toxins (nigericin and maitotoxin), endogenous danger signals like extracellular ATP, amyloid-β aggregates, uric acid crystals, or metabolic dysfunction, and pollutant particles as silica, asbestos, or alum (5, 82). The direct interaction between this broad range of activators and NLRP3 seems unlikely, and therefore it is suggested that NLRP3 is able to sense the cellular stress associated with the exposition to these agents. The precise molecular mechanism involved in the NLRP3 inflammasome activation remains elusive although recent studies begin to uncover the molecules and the cellular machinery responsible for this process (17, 83, 84).

Maintenance of ion gradients between different cellular compartments and between the cytosol and the extracellular environment is a feature of all living cells. Any alteration of this homeostasis will induce molecular mechanisms to respond and adapt to this aggression. Significant decrease of intracellular K^+^ is indeed detected during NLRP3 activation after the treatment with microbial pore-forming toxins or after P2X7 receptor engagement by extracellular ATP (85), where the hemichannel pannexin-1 plays a critical role (86). Interestingly, decrease of intracellular K^+^ is also detected during the NLRP3 inflammasome activation along with other sterile inductors as the decrease of osmolarity (3) or metabolic lipids (87), suggesting that intracellular K^+^ concentrations could be one of the common mechanisms involved in the activation of the NLRP3 inflammasome; however, its mechanism of function is not well understood (88–90).

In addition to the decrease of intracellular K^+^, a mobilization of Ca^+2^ in the cytosol is also detected in most of the stimulus that activates NLRP3. The ER is the main reservoir for intracellular Ca^+2^, and its mobilization as a consequence of the activation of inositol trisphosphate receptor has been observed during NLRP3 activation induced with different stimuli. The activation of P2X7 receptor also induces an influx of Ca^+2^ from the extracellular space; however, in this cellular context, the blockage of extracellular Ca^+2^ influx does not inhibit NLRP3 inflammasome, and artificial mobilization of Ca^+2^ is not sufficient to trigger the NLRP3 inflammasome activation in absence of K^+^ depletion (12, 14, 91). Cell swelling after hypotonic shock activates transient receptor potential cation channels (TRPM7 and TRPV2) involved in the modulation of intracellular Ca^+2^ that is crucial for the transforming growth factor beta-activated kinase 1 activation. These molecular events are required in combination with K^+^ efflux for NLRP3 inflammasome assembly (3). In addition, several works show evidences that extracellular Ca^+2^ can trigger mechanisms that activate inflammasome through G protein-coupled receptors (92, 93). The activation of these receptors leads to the mobilization of intracellular Ca^+2^ *via* phospholipase C activation with a concomitant reduction of cyclic AMP (cAMP) (92). The effect of this reduction in cAMP will be discussed later in the context of the negative regulation mechanisms of NLRP3. Interestingly, elevated concentrations of extracellular Ca^+2^ have been detected at infection sites or in ischemic injury, suggesting that extracellular Ca^+2^ would play a role as a DAMP (93).

Alteration of lysosomal function after phagocytosis of molecular crystals has been described as an additional activation process of NLRP3 inflammasome, possibly as a consequence of the activity of released lysosomal proteases altering the integrity of cellular organelles (94). Furthermore, other cellular stress associated with the intracellular ionic mobilization, as the induction of ER stress, is able to activate NLRP3 inflammasome in a K^+^ efflux-dependent manner. In this process, the endoribonuclease inositol-requiring enzyme 1α, an unfolded protein sensor expressed in ER, is required to activate the NLRP3 inflammasome (95, 96). Taken together, these data show that changes in intracellular ion concentration play a key role in the activation of NLRP3 inflammasome, although their precise molecular mechanism remains unclear.

Besides ion fluxes, changes in the cellular oxidative state is a common process detected during NLRP3 inflammasome activation, being mitochondrial damage one of the main source of reactive oxygen species (ROS) (97). Interestingly, several works link mitochondrial ROS production with changes in the intracellular concentration of K^+^ and Ca^+2^, which would induce depolarization of the mitochondrial membrane (91, 98). Mitochondrial ROS production has also been described as a novel NLRP3 activation mechanism involving a decrease of NADH levels after disruption of the glycolytic flux (82). Mitochondria have also been suggested as a cellular platform to assemble the NLRP3 inflammasome. The activation of NLRP3 induces its relocation from the ER to the proximity of the mitochondria in the perinuclear environment (97, 99). This recruitment requires the reorganization of the microtubule system (100). Moreover, the mitochondria may also release other molecules implicated in the activation of NLRP3 inflammasome as cardiolipin (101) or oxidized mitochondrial DNA (102, 103), and it has been shown that mitochondrial antiviral-signaling protein interacts with the PYD of NLRP3, being essential for their activation after the stimulation with ATP or nigericin but not with crystals (99). All these data point out the essential role of mitochondria in NLRP3 inflammasome activation.

Finally, caspase-4, and its mouse orthologous caspase-11, activates NLRP3 after recognition of cytosolic LPS (104, 105). This signaling is known as the non-canonical NLRP3 inflammasome activation pathway, and although the mechanism of caspase-4-inducing NLRP3 activation is not known, it is also dependent on the decrease of intracellular K^+^ (106–108).

## Regulatory Mechanisms of NLRP3 Inflammasome

**Figure 2:**
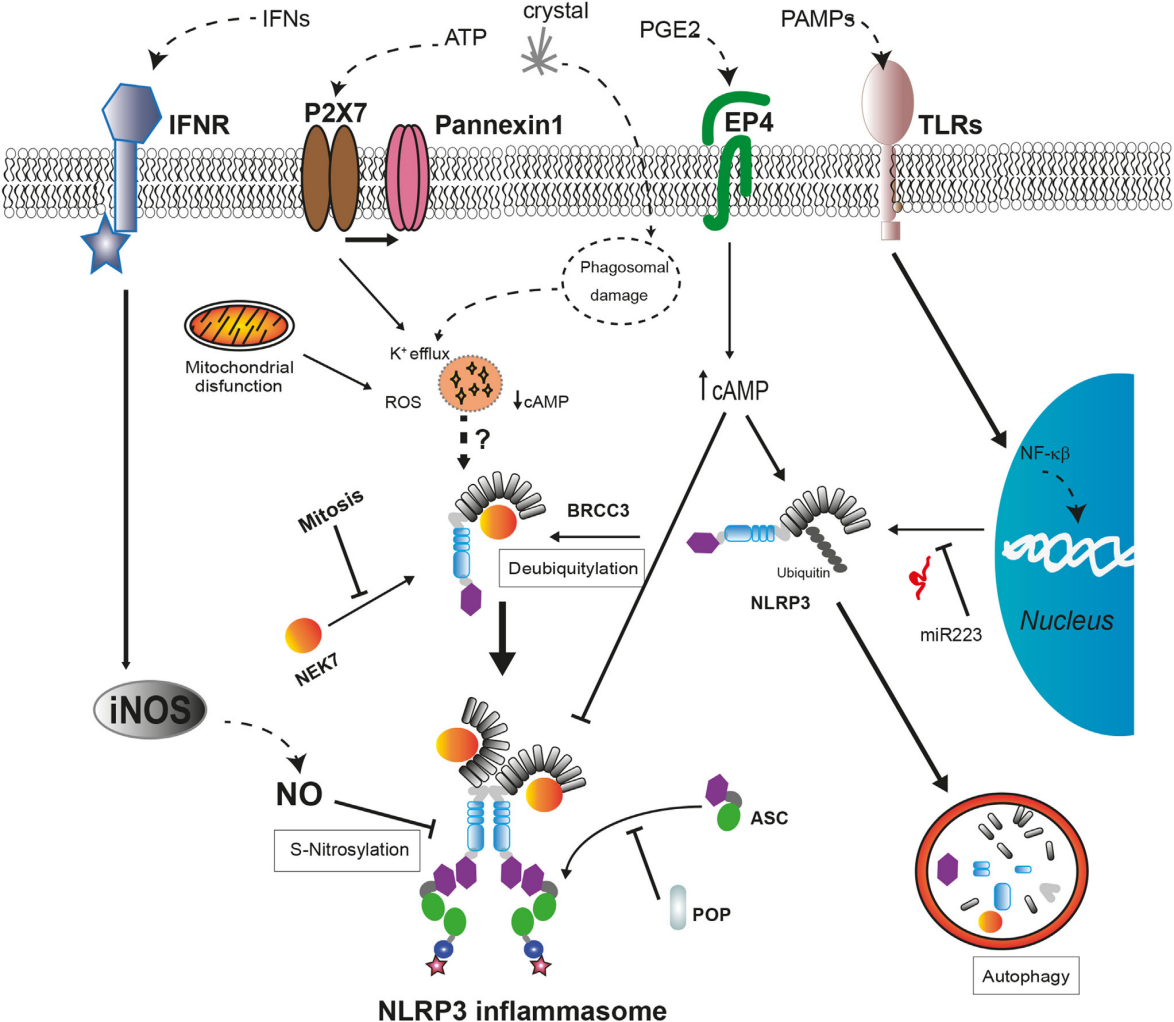
**Regulatory mechanisms of NLRP3 inflammasome assembly**. The expression levels of *NLRP3* are regulated by miR-223 in basal conditions but can be upregulated after cell recognition of pathogen-associated molecular patterns that induce NF-κB signaling pathway. The activation of NLRP3 by cellular damage signals as intracellular K^+^ decrease or reactive oxygen species (ROS) production requires a deubiquitylation of NLRP3 by BRCC3 and also their interaction with NEK7, only available during interphase. External signals, such as interferons (IFNs) or prostaglandin E_2_ (PGE2), negatively regulate NLRP3 through different mechanisms. The increase in nitric oxide (NO) produced by inducible nitric oxide synthase (iNOS) leads to the S-nitrosylation of NLRP3 impairing the assembly of the NLRP3 inflammasome. The increase of cyclic AMP (cAMP) induced by PGE2 signaling *via* prostaglandin E_2_ receptor 4 (EP4) activates the phosphorylation of NLRP3 reducing its oligomerization and increasing its ubiquitination to be degraded in autophagosomes. The interaction with pyrin domain-only proteins (POPs) can regulate NLRP3 inflammasome assembly by sequestering ASC or NLRP3.

Several proteins have been described as positive or negative regulators of the NLRP3 inflammasome assembly (Figure 2). Guanylate-binding protein 5 binds *via* its GTPase domain to the PYD of NLRP3 during inflammasome activation by most of the stimuli except crystalline agents. This interaction promotes the oligomerization of NLRP3 with ASC (109). Furthermore, several works have described that, during ATP stimulation, NLRP3 deubiquitination mediated by the Lys63-specific deubiquitinase BRCC3 is an early process essential for inflammasome activation (110–112).

Recent works have revealed a new NLRP3 inflammasome regulatory molecule, the never-in-mitosis A-related kinase 7 (NEK7), a serine, and threonine kinase required for mitosis progression (113). This protein interacts with the LRR domain of NLRP3 upstream of NLRP3 inflammasome assembly independent of their kinase activity (114). This interaction is required for NLRP3 inflammasome oligomerization and introduces a new component of inflammasome regulation, the restriction of NLRP3 inflammasome formation to cells in interphase (115). Moreover, the absence of NEK7 in cellular models harboring frequent CAPS-associated mutations in NLRP3 reduces their ability to activate caspase-1, while the association between NEK7 and mutant NLRP3 is stronger (114, 115). Further investigation is required to elucidate the role of NEK7 in the auto-activation of NLRP3 inflammasome in autoinflammatory syndromes.

Maintenance of low NLRP3 protein levels avoids the auto-assembly of NLRP3 inflammasome in the absence of a danger stimulus; therefore, transcriptional regulation of NLRP3 is an additional control mechanism to avoid unexpected inflammasome activation. Transcriptional regulation of NLRP3 requires NF-κB activation by TLR or IL-1 receptor type I (IL-1RI) signaling to increase NLRP3 protein concentration to certain level that can be activated after sensing a triggering stimulus (116, 117). Furthermore, the amount of NLRP3 mRNA is tightly regulated in myeloid cells through the microRNA miR-223, although this miRNA is not regulated by pro-inflammatory signals (118). In addition, under unstimulated conditions, NLRP3 is inhibited by posttranslational modifications with ubiquitin chains that also target NLRP3 for its degradation through proteasome or autophagy as will be described later (112). Other mechanism involved in the inhibition of the NLRP3 activity is the posttranslational modification of NLRP3 generated by the activation of inducible nitric oxide synthase. The increase of nitric oxide leads to the S-nitrosylation of NLRP3 impairing the assembly of the inflammasome, and this mechanism is suggested as a protective mechanism (119, 120). Therefore, the control of functional NLRP3 concentration within the cell is crucial for the activation of the inflammasome.

In addition to these negative regulatory mechanisms, two families of proteins containing CARD (COPs) or PYD (POPs) that could sequester either sensor proteins or effector proteins through PYD–PYD and CARD–CARD interactions have been described (121). In the absence of mutations, pyrin is also suggested to be a key regulator for the degradation of several inflammasome components (caspase-1, NLRP1, and NLRP3), preventing an excessive release of pro-inflammatory cytokines (122, 123). However, a recent work shows that the absence of pyrin in a mouse model leads to an increase in the release of IL-1β without affecting different inflammasome assembly (124). Therefore, the role of pyrin as an inflammasome inhibitor remains to be determined, and different domains among human and mouse pyrin proteins should be taken into account.

Cellular damage implicated in the activation of the NLRP3 inflammasome also activates autophagy, a mechanism involved in the clearance of intracellular pathogens and damaged organelles (102, 125). Autophagy is a negative mechanism to control the induction of the inflammatory response given its involvement in the degradation of damaged mitochondria, including molecular NLRP3 inflammasome inductors as mitochondrial DNA or ROS (102, 126, 127), the clearance of ASC specks (125), and pro-IL-1β (128). Ubiquitinated NLRP3 could be directed to the autophagosome for degradation by a complex with cAMP that recruits the E3 ubiquitin ligase MARCH7 (129, 130). This molecular mechanism can be triggered by activators of the adenylate cyclase as the neurotransmitter dopamine (130). Furthermore, an alternative negative regulatory mechanism has been described for NLRP3 inflammasome involving cAMP. The increase of cAMP induced by prostaglandin E_2_ signaling *via* prostaglandin E_2_ receptor 4 activates protein kinase A that phosphorylates NLRP3 in their NBD domain reducing its ATPase activity and oligomerization (131). Interestingly, this negative regulation could be disrupted by certain CAPS-associated mutations in the NBD of NLRP3 (114).

## Pyrin Inflammasome Activation Pathways and Regulatory Mechanisms

Recent data begin to unveil the mechanism involved in pyrin-inflammasome activation, as well as a protective mechanism concerned in blocking pyrin-inflammasome assembly. The inactivation of the RhoA GTPase by bacterial modification induces the activation of the pyrin inflammasome (73), suppressing a protective mechanism that avoids pyrin inflammasome activation through their downstream phosphorylation by serine/threonine-protein kinase N1 and N2 (132). This mechanism requires the phosphorylation of certain amino acids of pyrin (S208 and S242 in human) allowing their binding to regulatory protein 14-3-3 and blocking the formation of the pyrin inflammasome (51, 132). Pyrin inflammasome activation through bacterial toxins is detected in human and mice, indicating that the C-terminal B30.2/SPRY domain, present only in human, is not required for their activation (133). Interestingly, this domain harbors most of the mutations detected in familial Mediterranean fever (FMF) patients, although some mutations affect one of the serine, in other domain of the protein, described as a key amino acid in the protective mechanism against the uncontrolled activation of pyrin inflammasome (see below). The inhibition of microtubule polymerization by colchicine abolishes pyrin inflammasome assembly induced by bacterial toxins, without affecting pyrin dephosphorylation (133, 134). However, this control of pyrin inflammasome activation leaded by microtubules is not effective in FMF patients that harbor mutations in B30.2/SPRY domain (133), suggesting that these mutations may force a protein conformation that aids the assembly of pyrin inflammasome after their dephosphorylation.

## Inflammasome-Associated Secretome

The formation of inflammasome leads to the activation of caspase-1, and this protease triggers a broad number of cellular events as a consequence of its catalytic activity, including the release of cytosolic proteins associated with a specific type of cell death termed pyroptosis. Specifically, the analysis of the secretome associated with caspase-1 has revealed the key role of this protease in the unconventional secretion of multiple essential molecules involved in the inflammatory process. From them, the cytokine IL-1β is one of the most prominent and critical products of inflammasome activation, since it is a key regulator of the inflammatory response and is the most important current target of therapeutic treatments for autoinflammatory syndromes. The synthesis of IL-1β mRNA and the production of the inactive precursor form of IL-1β are strongly induced by microbial products such as LPS signaling *via* TLR or by IL-1 itself signaling through the IL-1RI (135). Caspase-1 is able to process the inactive precursor form of IL-1β to its mature bioactive form and induce its release. Similarly, caspase-1 is also responsible to the maturation of the inactive IL-18 precursor, another IL-1 family cytokine, to its bioactive form (136).

Both IL-1β and IL-18 are the canonical cytokines signaling downstream inflammasome activation, but beyond these caspase-1 substrates, caspase-1 also controls the unconventional release of other cytosolic proteins (FGF-2, thioredoxin, and annexins), lysosomal proteins (cathepsins and cystatins), or nuclear proteins (HMBG1, IL-1α) that are not direct substrates of the protease (13, 14). In addition, caspase-1 also controls the release of large complex protein aggregates as ASC inflammasome oligomers that are now able to spread caspase-1 activation to adjacent cells and maintain inflammasome signaling (11, 15).

Unconventional protein release induced by caspase-1 has been widely studied for IL-1β, a cytokine that does not follow the conventional route of protein secretion through ER or Golgi. Different mechanisms of unconventional secretion have been explored to explain this process including the release through exocytosis of secretory lysosomes (137) or extracellular vesicles released after NLRP3 inflammasomes activation (138, 139). Caspase-1-induced pyroptosis is associated with an increase in plasma membrane permeation that may help a passive release of IL-1β (140). The destabilization of cell membrane integrity during pyroptosis is induced by the cleavage of the cytosolic substrate gasdermin D by caspase-1 or caspase-4; gasdermin D N-terminus integrates into the plasma membrane forming pores (141–143). The application of membrane stabilizing agents, as the complex polyphenol punicalagin, prevents the execution phase of pyroptosis and release of mature IL-1β from macrophages after NLRP3 inflammasome activation, suggesting that loss of plasma membrane is involved in this secretion in parallel with cell death (144). Interestingly, the stabilization of the plasma membrane by punicalagin inhibits the release of IL-1β in neutrophils in the absence of cell death (144). Therefore, the secretion of bioactive form of IL-1β requires membrane permeation and may occur in secreting cells as neutrophils where NLRP3 inflammasome does not induce pyroptosis (145, 146). Initially, these mechanisms are not mutually exclusive and may participate in the secretion of IL-1β depending on the intensity of the stimulus and cell type. The release of other caspase-1-dependent secretome proteins is less studied, and the involvement of pyroptotic cell death in this process is not known, neither its contribution to the pathophysiology of autoinflammatory syndromes.

## Implications of NLRP3 Inflammasome in CAPS

Cryopyrin-associated periodic syndromes are rare hereditary autosomal-dominant autoinflammatory diseases with an estimated prevalence of 1–3 cases per million of inhabitants (147, 148). They include familial cold urticaria syndrome (FCAS) (59), Muckle–Wells syndrome (MWS) (149), and chronic infantile neurological cutaneous and articular (CINCA) syndrome also known as neonatal onset multisystemic inflammatory disease (NOMID) (150). All three syndromes were independently described and latterly found to be caused by gain-of-function mutations in the *NLRP3* gene, located in the short arm of chromosome 1 (1q44) (37, 38). Mutant NLRP3 drives a constitutive hyperactivity of inflammasome, activation of caspase-1, and an excessive unregulated release of IL-1β, although systemic circulating levels of IL-1β during inflammatory flares are in most cases undetectable (11, 147).

Clinical features of CAPS are related to systemic effects of IL-1β-inducing fever, malaise, fatigue, and chronic pain along with a blood serum rise of acute-phase reactants, such as C-reactive protein and serum amyloid A. CINCA/NOMID is characterized for an almost continuous early onset inflammatory state with fever and non-pruritic migratory urticaria-like rash; central nervous system symptoms and arthropathy are common. MWS shows a moderate phenotype with latter onset of fever, rash, arthralgia, conjunctivitis, uveitis, sensorineural deafness, and potentially life-threatening amyloidosis. FCAS is a milder familial condition characterized by febrile urticarial rash with headache, arthralgia, and sometimes conjunctivitis but no central nervous system symptoms and is typically triggered by cold exposure. FCAS, MWS, and CINCA/NOMID are considered a clinical continuum than distinct diseases as intermediate phenotypes occur; being FCAS the mildest and CINCA/NOMID the most severe forms (151). Neurologic manifestations in CAPS are common including headache, sensorineural hearing loss, myalgia, chronic aseptic meningitis, increased intracranial pressure, cerebral atrophy, seizures, and mental retardation (152). Musculoskeletal symptoms in CAPS are also very frequent; up to 86% may have any musculoskeletal manifestation during follow up, 30% at onset. In a large cohort, these included arthralgia in 86% and arthritis in 58%; joint destruction and typical knee deformities appeared rarely (9% and 2%, respectively). Tendinopathies occurred in 21.5%, tender points in 16.5%, and myalgia in 33% of patients (153).

To date, a total of 177 variants of *NLRP3* gene have been included in infevers database (39), most of them located in exons 3 or 4 and intron 4. Among them, the most frequent is R260W (40, 148) and are associated with a milder phenotype along with A439V. The variants, T348M and D303N, and low frequency mutations are associated with a severe phenotype; E311K accounts for a high rate of hearing loss. On the other hand, Q703K or V198M variants have little clinical significance and are considered a functional polymorphism and a low penetrance variant, respectively. Clinically affected patients with no germline mutations could have an NLRP3 somatic mosaicism (154–156). The highly heterogeneous phenotypes within identical genotypes show the need for advancing the underlying understanding of the pathophysiological mechanisms.

The relevance of NLRP3 mutations as key players in the induction of these autoinflammatory syndromes has been explored in animal models. Specifically, the development of knock-in mouse strains harboring some of the frequent mutations detected in CAPS syndrome has demonstrated the pivotal role of IL-1β and the innate immunity in the pathogenesis of this syndrome; meanwhile, the adaptive immune system seems not to be involved (41). These animal models exhibit an autoinflammatory disease similar to that in humans associated with an inflammasome hyperactivation and unregulated release of IL-1β (41–43). Humanized mice expressing CAPS-associated mutation D305N present an increased sensitivity to endotoxin and develop progressive and debilitating arthritis (44). Furthermore, the study of these knock-in animal models has revealed the critical role of microbiota as inducer of disease, and myeloid and mast cells as cellular sources of IL-1β in the development of the skin inflammation (42). In addition, the study of these CAPS-like animals has revealed the key role of IL-18 in the early tissue inflammation and suggests the presence of other players beyond IL-1β and IL-18 that are involved in inflammatory activities associated with the pyroptosis and possible by the caspase-1-associated secretome (45). A recent study with blood monocytes from patients affected by CAPS detected a high level of cellular stress including elevated levels of ROS compared with healthy subjects (157). Interestingly, associated with this oxidative stress, there is a reduction in the production of the anti-inflammatory cytokine, IL-1 receptor antagonist (IL-1Ra) (158), as well as a decrease in the threshold of inflammasome activation of CAPS monocytes (159). The exposure of this monocyte to inflammatory stimuli such as LPS induces an increase in the release of ATP that produces an increase in the secretion of IL-1β, IL-18, and IL-1α (159). These data collectively suggest the involvement of genetic and environmental factors beyond a single mutation that needs to be explored to obtain a more accurate clinical picture of this disease.

## The Pyrin Inflammasome and Implications in FMF and Pyrin-Associated Autoinflammation with Neutrophilic Dermatosis (PAAND)

The inflammasome sensor protein pyrin is primarily expressed in myeloid cells, and wild-type pyrin negatively modulates NLRP3 inflammasome-dependent IL-1β release (160). However, mutations in the *MEFV* gene (that codify for pyrin) are associated with two clinically different autoinflammatory syndromes: FMF and PAAND (51); in both diseases, mutated pyrin associates with high serum IL-1β levels during febrile episodes.

Familial Mediterranean fever is the most common inherited monogenic autoinflammatory disease worldwide and is caused by loss-of-function mutations in *MEFV* gene, mostly affecting eastern Mediterranean population (161). Classically considered an autosomal recessive condition, it is actually discussed if it should be considered an autosomal-dominant disease with variable penetrance, since heterozygosis mutations are associated with clinical autoinflammatory FMF manifestations (162). Nevertheless, homozygosis is associated with severe FMF phenotypes.

Familial Mediterranean fever patients typically show recurrent self-limited acute febrile attacks of 1–3 days of duration, accompanied by inflammation of serosa and/or synovial linings (90% abdominal pain, 40% arthritis, and 30% thoracic pain), myalgia (40%), and erysipeloid type rash (20%). Onset before the age of 18 is common and has been associated with higher rates of arthritis, arthralgia, myalgia, and erysipeloid-like rash (163). Pleuritis, pericarditis, scrotal pain, aseptic meningitis, thrombosis, and vasculitis may appear during flares, but FMF can also be associated with many other disorders in a non-canonical manner (164). The most severe complication of FMF is amyloidosis as a result of chronically uncontrolled inflammation that occurs in undiagnosed or untreated patients; it is more likely to occur in patients with recurrent arthritis (165). Renal amyloidosis seldom occurs as the first clinical manifestation of FMF, and these individuals are referred as phenotype II patients (166). Homozygosis in serum amyloid A gene 1 (alpha/alpha) and male sex have shown influence on the risk of developing amyloidosis in FMF patients (167, 168).

Over 300 *MEFV* gene variants have been described (39), but only 14 occur frequently in FMF (E148Q, E167D, T267I, P369S, F479L, I591T, M680I, I692del, M694I, M694V, K695R, V726A, A744S, and R761H). The majority of pathogenic mutations are located in exon 10, being M694V the most frequent *MEFV* mutation encountered in FMF patients; its presence in homozygosis or compound heterozygosis is related to severe phenotype. In exon 2, E148Q is the most frequent *MEFV* variant in asymptomatic carriers and in some population subsets, it may even be a benign polymorphism (47); it is present in FMF patients with a mild phenotype (48). Diagnosis of FMF is sometimes elusive and is made under clinical basis. Validated diagnostic criteria include typical clinical manifestations, family history, and response to colchicine therapy (169). Genetic testing leads to higher rates of diagnosis (170, 171), supporting but not excluding clinical diagnosis (172). Inconsistency among similar phenotypes may be explained by major histocompatibility class I chain-related gene A alleles, as shown in a study on homozygous M694V population (173).

Pyrin-associated autoinflammation with neutrophilic dermatosis is an inherited autosomal-dominant autoinflammatory disease characterized by childhood onset. This autoinflammatory syndrome is characterized by recurrent episodes of neutrophilic dermatosis, fever, elevated acute-phase reactants, arthralgia, and myalgia or myositis (51). PAAND is caused by a loss in guard mechanism of pyrin due to S242R mutation that leads to a non-phosphorylated pyrin in S242 (51). Dephosphorylated pyrin loses interaction with the protein 14-3-3 and thus forming a constitutive active inflammasome by recruiting ASC (51). Mutations and clinics of PAAND are distinct from FMF because of a clearly dominant inheritance pattern and for its longer fever episodes (lasting weeks), more prominent cutaneous features, and absence of serositis or amyloidosis (51). Currently, it is not fully understood how mutations in two regions of the same protein can induce different diseases. FMF-related mutations have recently been found to induce a pyrin-inflammasome that could be dephosphorylated by RhoA GTPase and not inhibited by colchicine, questioning the critical dependency on microtubules for ASC aggregation and inflammasome activation (133). PAAND-associated mutations in MEFV gene are associated with a reduction in the binding of pyrin to microtubules, decreasing the threshold to assemble pyrin inflammasome. It is not known if PAAND syndrome-associated pyrin inflammasome is dependent on microtubules, although the use of colchicine has shown partial clinical benefit in this patient (51). Cutaneous manifestations of PAAND resemble other autosomal-dominant monogenic autoinflammatory disease called pyogenic arthritis, pyoderma gangrenosum, and acne (PAPA) syndrome, in which arthritis is the distinct and prominent feature. PAPA is caused by mutations at proline–serine–threonine phosphatase-interacting protein 1 gene (174). This protein is a cytoskeleton-associated adaptor protein that interestingly binds pyrin and regulates IL-1β production (175). The generation of knock-in mice with FMF-associated pyrin mutation (harboring a human C-terminal B30.2/SPRY domain that is absent in mouse *Mefv* gene) has shown data supporting the activation of a pyrin-inflammasome and an increase of IL-1β in this animal model independent of NLRP3 (49). Furthermore, autoinflammation in this animal model is dependent on the ASC-caspase-1 axis and IL-1β, whereas IL-1α and caspase-8 are dispensable for the inflammation observed in this FMF model (50).

## Current Therapeutics Targeting the Inflammasome Pathway

Inflammasomes are main drivers of autoinflammatory diseases as well as important regulators of innate immunity and inflammation. Although specific drugs that directly interfere with inflammasome activation are under development, current treatments used in clinic target upstream regulation process, in the case of colchicine, or downstream IL-1 signaling (176).

Colchicine is the classical mainstay treatment for FMF (177), decreasing attack frequency, improving quality of life, and preventing amyloidosis (178, 179). Clinical response to colchicine is considered a supportive diagnostic criterion for FMF, but it shows no benefit in CAPS patients. Colchicine is known to directly recover activity of the GTPase RhoA and therefore suppresses pyrin oligomerization but is also able to interfere with neutrophil migration and adhesion by downregulating the expression and distribution of selectins on neutrophils and endothelial cells (180). Interestingly, pyrin associates with microtubules and colocalizes with actin filaments (181). Thus, colchicine treatment may also prevent cytoskeletal changes that favor pyrin inflammasome assembly. However, recent data have shown that microtubule polymerization is not a requirement for pyrin inflammasome activation in FMF patients in contrast with wild-type pyrin carriers, providing a new concept for understanding the molecular mechanisms present in the activation of pyrin inflammasome (133). Nevertheless, some FMF patients are resistant to colchicine, and in this subset of patients, IL-1 blocking agents have shown efficacy (182–184). Anakinra therapy was also effective in a patient diagnosed with PAAND (51).

As exposed above, IL-1β is one of the main products of inflammasome and caspase-1 activation and exerts its inflammatory action by binding to the IL-1RI (185), this binding is antagonized by the IL-1Ra, a protein that binds IL-1RI without agonistic activity preventing IL-1β binding and signaling (185).

Therapies blocking IL-1 are available for the treatment of CAPS and other autoinflammatory syndromes (i.e., colchicine-unresponsive FMF patients). Anakinra is the recombinant form of IL-1Ra and was the first anti-IL1 agent clinically available. Due to its short half-life, it has to be administered by subcutaneous injection daily, and side effects are common at the site of injection; also liver enzymes need to be monitored regularly. There is a strong evidence of the effectiveness of anakinra for CAPS treatment (186, 187), with improvement of clinical features like hearing loss or amyloidosis with quick relapse of symptoms after withdrawal, demonstrating the requisite of daily injections in persistent and severe phenotypes. Despite its effectiveness, sore daily injections of anakinra are sometimes unpopular among patients, and in selected cases with mild phenotypes are possible to use it on demand basis during inflammatory attacks as in other autoinflammatory diseases (188, 189).

Other anti-IL-1 agents have been developed with a better pharmacokinetic profile and are actually approved for the treatment of CAPS. Canakinumab is a humanized monoclonal antibody against IL-1β administered intravenously or subcutaneously at a dose of 2–4 mg/kg every 8 weeks, and it is licensed for treatment of CAPS patients over 4 years of age. It has shown a very rapid and sustained response with little side effects, mainly infections, with stabilization of the majority of sequelae and potential improvement in clinical manifestations such as sensory-neural hearing loss (190). Abnormal bone formation in CAPS patients is unaffected by IL-1 blockage (191), revealing that other pathways downstream NLRP3 inflammasome play important roles in the clinical manifestations. Canakinumab up-titration may be needed and is actually encouraged in partial responders and severe phenotypes, rising the dose and shortening administration up to 8 mg/kg every 4 weeks (192).

Rilonacept is an engineered IL-1 trap that neutralizes circulating IL-1β and IL-1α, and it is administered subcutaneously with a load dose of 320 mg followed by 160 mg weekly (185). After initial pilot study and phase III studies, rilonacept was the first drug approved for treatment of CAPS, including FCAS and MWS in children of 12 years and older, due to its safety and effectiveness (193, 194). Benefits were obtained within hours of its administration with maximal effect within day 6 and 10, with mild or moderate adverse reactions.

Caspase-1 activation precedes IL-1β release after inflammasome activation; therefore, there have been advances in generating specific and clinical relevant caspase-1 inhibitors. The most developed caspase-1 inhibitor for therapeutic use is VX-765, an orally available pro-drug that is rapidly hydrolyzed by plasma and liver esterase into a potent and selective inhibitor of caspase-1 (195). In fact, VX-765 was able to reduce the release of IL-1β and IL-18 in monocytes of patients with FCAS treated with LPS (196). However, its clinical use is still under investigation.

The standard goal to treat autoinflammatory syndromes, specially CAPS patients, will be to directly target NLRP3 inflammasome using small compounds, in this respect a compound developed by Pfizer (CP-456773 or CRID3, recently renamed as MCC950) has been proved to block IL-1β release in CAPS monocytes after LPS treatment, being able to reduce clinical symptoms in an animal model of CAPS (197, 198). Furthermore, this compound has been recently found to reduce inflammation in animal models of renal, dermal, and pulmonary inflammation (199, 200). Therefore, CP-456773 represents a promising drug for the treatment of autoinflammatory syndromes.

## Conclusion

Mutations in genes coding for inflammasome sensor proteins, such as NLRP3 or pyrin, accomplish for the development of different autoinflammatory diseases by uncontrolled activation of caspase-1 and the aberrant release of pro-inflammatory cytokines. In physiological conditions, the inflammasome pathway is activated in response to dangerous situations provoked by infections, tissue injury, or cellular stress, being the inflammasome formed by the sensor NLRP3 the most promiscuous inflammasome pathway activated in many different situations. Furthermore, non-mutated NLRP3 activation has been involved in different autoinflammatory syndromes, and, for example, patients with mutations in *PLCG2* (autoinflammation and phospholipase Cγ2-associated antibody deficiency and immune dysregulation, APLAID syndrome) present an aberrant cytosolic Ca^+2^ signaling leading to NLRP3 activation, or patients with mutations in the deubiquitinase *OTULIN* (otulipenia) result in aberrant IL-1 production by NLRP3 activation (201, 202). NLRP3 has also been implicated as a key inflammasome sensor protein in different chronic diseases; in these circumstances, different endogenous danger signals activate NLRP3 and could contribute to the inflammatory response in metabolic and degenerative diseases, such as gout, type 2 diabetes, obesity atherosclerosis, or Alzheimer’s disease (6, 203, 204). Therefore, inflammasome is central in autoinflammatory diseases, and increasing our understanding on NLRP3 and pyrin activation may lead to development of more potent novel therapies for the treatment of not only autoinflammatory syndromes but also for chronic inflammatory, metabolic, and degenerative diseases.

## Author Contributions

CT-M: figure preparation. CT-M, PC, and PP: literature search and manuscript preparation.

## Conflict of Interest Statement

The authors declare that the research was conducted in the absence of any commercial or financial relationships that could be construed as a potential conflict of interest. The reviewer MJ and handling Editor declared their shared affiliation, and the handling Editor states that the process nevertheless met the standards of a fair and objective review.
